# Structure–mechanical property correlations in mechanochromic luminescent crystals of boron difluoride dibenzoylmethane derivatives

**DOI:** 10.1107/S2052252515015134

**Published:** 2015-09-22

**Authors:** Gamidi Rama Krishna, Ramesh Devarapalli, Rajesh Prusty, Tiandong Liu, Cassandra L. Fraser, Upadrasta Ramamurty, Chilla Malla Reddy

**Affiliations:** a Department of Chemical Sciences, Indian Institute of Science Education and Research (IISER) Kolkata, Mohanpur Campus, Mohanpur 741252, India; b Department of Materials Engineering, Indian Institute of Science, Bangalore 560012, India; c Department of Chemistry, University of Virginia, Charlottesville, Virginia 22904, USA; d Center of Excellence for Advanced Materials Research, King Abdulaziz University, Jeddah 21589, Saudi Arabia

**Keywords:** crystal engineering, intermolecular interactions, mechanochromism, mechanical properties, nanoindentation, organic solid-state reactions, hydrogen bonding

## Abstract

The structure and mechanical properties of crystalline materials of three boron difluoride dibenzoylmethane (BF_2_dbm) derivatives were investigated to examine the correlation, if any, among mechanochromic luminescence (ML) behaviour, solid-state structure, and the mechanical behaviour of single crystals. Qualitative mechanical deformation tests show that the crystals of BF_2_dbm(^*t*^Bu)_2_ can be bent permanently, whereas those of BF_2_dbm(OMe)_2_ exhibit an inhomogeneous shearing mode of deformation, and finally BF_2_dbmOMe crystals are brittle. Quantitative mechanical analysis by nano­indentation on the major facets of the crystals shows that BF_2_dbm(^*t*^Bu)_2_ is soft and compliant with low values of elastic modulus, *E*, and hardness, *H*, confirming its superior suceptibility for plastic deformation, which is attributed to the presence of a multitude of slip systems in the crystal structure. In contrast, both BF_2_dbm(OMe)_2_ and BF_2_dbmOMe are considerably stiffer and harder with comparable *E* and *H*, which are rationalized through analysis of the structural attributes such as the intermolecular interactions, slip systems and their relative orientation with respect to the indentation direction. As expected from the qualitative mechanical behaviour, prominent ML was observed in BF_2_dbm(^*t*^Bu)_2_, whereas BF_2_dbm(OMe)_2_ exhibits only a moderate ML and BF_2_dbmOMe shows no detectable ML, all examined under identical conditions. These results confirm that the extent of ML in crystalline organic solid-state fluorophore materials can be correlated positively with the extent of plasticity (low recovery). In turn, they offer opportunities to design new and improved efficient ML materials using crystal engineering principles.

## Abstract 2

Structure–mechanical property studies confirm that the extent of mechanochromic luminescence in crystalline organic fluorophores positively correlates with the extent of plasticity.

## 1. Introduction   

Organic solid-state fluorophore materials exhibit considerable promise in applications such as light-emitting diodes (Strassert *et al.*, 2011 ▸; Friend *et al.*, 1999 ▸), lasers (Gao *et al.*, 2010 ▸; Schmidtke *et al.*, 2002 ▸), two-photon fluorescent materials (Denk *et al.*, 1994 ▸) and in opto-electronic devices (Yoon *et al.*, 2010 ▸). However, detailed understanding of the mechanism(s) behind their properties is essential before they can be successfully deployed. The mechanochromic luminescence (ML) in their solid state is mainly due to the chemical or physical structural changes, for example, the latter involves the reorganization of molecules by changes in conformation, relative position of the molecules, intermolecular interactions or all of the processes together, upon subjecting them to stress (Krishna *et al.*, 2013 ▸; Zhang *et al.*, 2010 ▸; Anthony *et al.*, 2010 ▸). Usually, the solid-state reorganization of molecules is closely related to the mechanical properties of their crystals, because both the properties depend intricately upon the intermolecular interactions (Reddy, Padmanabhan *et al.*, 2006 ▸; Reddy, Kirchner *et al.*, 2006 ▸; Ghosh & Reddy, 2012 ▸; Reddy *et al.*, 2010 ▸; Sun & Hou, 2008 ▸; Feng & Grant, 2006 ▸). The design of new molecular materials with target mechanical properties requires precise control over the weak intermolecular interactions in the structure because their strength and directionality play a crucial role in the deformation process (Ghosh *et al.*, 2013 ▸, 2015 ▸). A number of studies show that the changes in solid-state luminescence can be brought about through either molecular design or by changes in crystal structure (Perruchas *et al.*, 2010 ▸; Yoon *et al.*, 2010 ▸; Kozhevnikov *et al.*, 2008 ▸; Zhang *et al.*, 2010 ▸). However, almost nothing is known about how one can control ML behaviour through the *engineering of solid-state packing in crystals*, which is a considerable challenge as the precise control of weak intermolecular interactions is nontrivial (Desiraju & Steiner, 1999 ▸; Desiraju, 1997 ▸, 2005 ▸).

**Figure 1:**
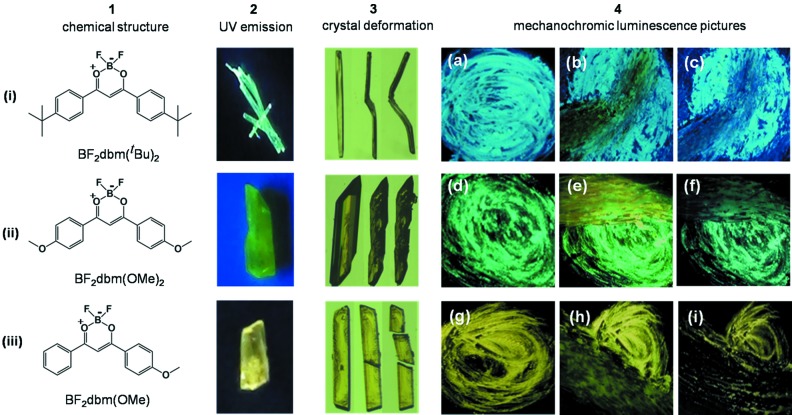
Chemical structures (i), (ii) and (iii) of the compounds (1), molecular crystals of the three individual compounds illuminated under UV (2), their qualitative deformation behaviour upon mechanical action (3), and mechanochromic luminescence behaviour of the crystals upon smearing (4). In column 4, (*a*), (*d*) and (*g*) show the UV (365 nm) emission colour of initial powder films prepared by gently grinding single crystals of the three samples using a mortar and pestle. Images (*b*), (*e*) and (*h*) correspond to the films after firmly scratching the initial films with a pestle, resulting in a colour change from cyan to yellow for BF_2_dbm(^*t*^Bu)_2_ and green to yellow for BF_2_dbm(OMe)_2_ while no colour change was observed for BF_2_dbmOMe at room temperature (*h*), respectively. For both BF_2_dbm(^*t*^Bu)_2_ and BF_2_dbm(OMe)_2_ compounds, the films recover to the parent colour shown in (*c*) and (*f*) after a few minutes of heating with a hot-air gun. In the case of BF_2_dbmOMe the ML experiments were repeated at −98°C by immersing the mortar into frozen methanol by cooling wth liquid N_2_ (*g*), but no significant colour change was observed in (*h*) and (*i*).

In recent work we have shown that the easy-to-deform polymorph of a BF_2_AVB derivative shows better ML behaviour, in terms of reversibility, compared with its brittle polymorph (near-instantaneous recovery) (Krishna *et al.*, 2013 ▸). The presence of slip planes in the former, which facilitate plastic deformation through shear sliding, was suggested as the reason for the prominent reversible ML. This observation, in turn, indicates that the crystal engineering of ML materials should embody the key design principle of introducing slip planes in the crystal packing. It is important to note here that such principles are not only useful for ML materials, but also in other instances such as for improving the tabletability of pharmaceutical solids (Krishna *et al.*, 2015 ▸; Bag *et al.*, 2012 ▸; Karki *et al.*, 2009 ▸; Jain, 1999 ▸; Chattoraj *et al.*, 2010 ▸), for the design of flexible optoelectronic crystals (Briseno *et al.*, 2006 ▸; Ruiz *et al.*, 2012 ▸; Minemawari *et al.*, 2011 ▸), flexible waveguides (Chandrasekhar & Chandrasekar, 2012 ▸; Chandrasekar, 2014 ▸; Balzer *et al.*, 2003 ▸; Drain, 2002 ▸; Lehn, 2002 ▸) *etc*. In trying to further such knowledge, the present work examines the crystal structures, mechanical properties and ML in BF_2_dbm(^*t*^Bu)_2_, BF_2_dbm(OMe)_2_ and BF_2_dbmOMe (Fig. 1 ▸) compounds, with a view to determining if any correlation exists amongst these three attributes. The mechanical behaviour of single crystals of the respective compounds was evaluated qualitatively as well as quantitatively. The former was accomplished by manually applying mechanical stress and deforming crystals using a pair of metal forceps and a metal needle while observing the crystal under a microscope, whereas the nanoindentation (NI) technique was utilized for quantitative property measurements. The measurements made and bulk ML properties are rationalized on the basis of the presence or absence of active slip planes in the respective crystal packing. In addition to both qualitative and quantitative analysis, we employ the Hirshfeld two-dimensional fingerprint plot analysis to quantify the dominant hydrogen bonding functionalities present in the three compounds (Fig. S5) (McKinnon *et al.*, 2007 ▸).

Slip planes (weak interaction planes) are generally found in molecular crystals when the weakly interacting functional groups such as ^*t*^Bu, —OMe, —SMe, —Cl *etc.* are organized in such a way that the molecules interact only *via* dispersive and nonspecific van der Waals (vdW) interactions across a crystallographic plane. For this study, the molecular derivatives of boron difluoride dibenzoylmethane (BF_2_dbm) were selected for the following reasons: (i) they have the ability to absorb ultraviolet (UV) light over a wider range of wavelengths than many other organic sunscreen agents; (ii) they form stable crystalline complexes with boron trifluoride, which are highly fluorescent under UV irradiation; (iii) high sensitivity of the emission colour to the conformational changes. With this in mind, we introduced the hydrophobic groups such as ^*t*^Bu, —OMe as substituents to promote the formation of active slip planes in the crystal packing, which promote plastic deformation leading to the creation of low energy defects. In turn, we expect that the fluorophore crystals with higher levels of plasticity can exhibit reversible ML behaviour (Krishna *et al.*, 2013 ▸; Anthony *et al.*, 2010 ▸; Reddy, Kirchner *et al.*, 2006 ▸; Reddy, Padmanabhan *et al.*, 2006 ▸; Sun & Hou, 2008 ▸).

## 2. Results and disscussion   

All three compounds were synthesized by following known procedures (Karasev & Korotkich, 1986 ▸; Yoshii *et al.*, 2013 ▸; Sun *et al.*, 2012 ▸; Zawadiak & Mrzyczek, 2012 ▸). Single crystals of the three compounds BF_2_dbm(^*t*^Bu)_2_, BF_2_dbm(OMe)_2_ and BF_2_dbmOMe, prepared by the standard slow evaporation method, were utilized for X-ray structure determination as well as for the mechanical property studies. Among the three compounds, crystals of BF_2_dbm(^*t*^Bu)_2_ are cyan in colour (450 nm), BF_2_dbm(OMe)_2_ green (498) and BF_2_dbmOMe yellow (554), as shown in Fig. 1 ▸. The initial qualitative mechanical deformation tests confirmed that molecular crystals of BF_2_dbm(^*t*^Bu)_2_ and BF_2_dbm(OMe)_2_ undergo plastic bending and shearing deformation, respectively, whereas BF_2_dbmOMe was found to be brittle under the test conditions. Crystals of both BF_2_dbm(^*t*^Bu)_2_ and BF_2_dbm(OMe)_2_ materials exhibited prominent ML properties when the powders of the respective crystals were scratched firmly using a mortar and pestle at room temperature, but no detectable ML behaviour was noticed in case of the brittle crystals of BF_2_dbmOMe under similar test conditions.

## 3. Crystal structure analysis   

### 3.1. BF_2_dbm(^*t*^Bu)_2_   

**Figure 2:**
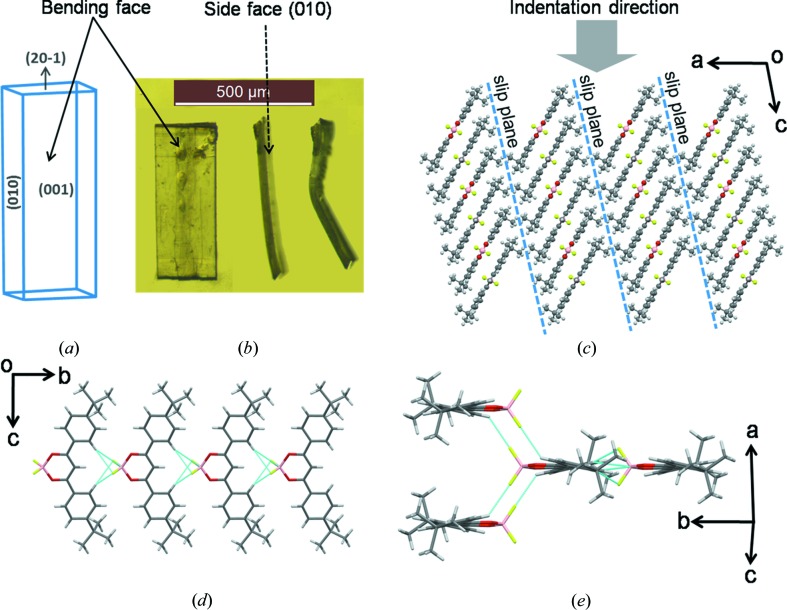
Crystal packing of BF_2_dbm(^*t*^Bu)_2_. (*a*) Schematic diagram of the habit planes or face indices. (*b*) Distinct faces (major and side faces) of the original crystal and the bent crystal to visualize the bending face. (*c*) Showing the indentation direction and representation of slip planes formed *via* hydrophobic *tert*-butyl groups in BF_2_dbm(^*t*^Bu)_2_ crystal packing. (*d*) Head-to-tail interaction of molecules *via* C—H⋯(BF_2_O_2_), which are further linked *via* C—H⋯F interactions to form two-dimensional sheets.

BF_2_dbm(^*t*^Bu)_2_ crystallizes in the centrosymmetric monoclinic space group *C*_2_/*c*, with half the molecule in an asymmetric unit (Fig. S1(i), for clarity the full molecule has been shown). Since there are no conventional hydrogen-bonding functional groups, the molecular packing is dominated mainly by weak C—H⋯F interactions. Plenty of examples are available in the literature on the utilization of C—H⋯O and C—H⋯F intermolecular interactions for crystal engineering (Hathwar *et al.*, 2011 ▸; Thalladi *et al.*, 1995 ▸; Schönleber *et al.*, 2014 ▸; Thakur *et al.*, 2010 ▸). Thalladi *et al.* suggested that C—H⋯F interactions can also be as important as C—H⋯O and C—H⋯N hydrogen bonds for stabilizing the specific crystal structures (Thalladi *et al.*, 1998 ▸; Dunitz & Schweizer, 2006 ▸; Chopra & Row, 2011 ▸). In the present case, the bifurcated C—H⋯F (*d*/Å, θ/°; 2.56 Å, 167.36°) and C—H⋯B (3.058 Å, 160.76°) interactions between the phenyl and BF_2_O_2_ groups connect molecules along the *b*-axis in a head-to-tail fashion (Fig. 2 ▸ *d*) (Alemany *et al.*, 2014 ▸), which are further linked along the *c*-axis *via* an additional C—H⋯F (2.46 Å, 135.98°) interaction to form thick two-dimensional sheets as shown in Fig. 2 ▸(*e*). The two-dimensional sheets pack together *via* the close packing of hydrophobic ^*t*^Bu groups resulting in slip planes or weak interaction planes parallel to (100) in the crystal packing (Fig. 2 ▸ *c*). The slip planes exist orthogonal to comparatively strong C—H⋯F interactions. Therefore, the overall crystal packing is anisotropic and hence promotes plasticity in the crystals (Reddy, Kirchner *et al.*, 2006 ▸; Reddy, Padmanabhan *et al.*, 2006 ▸; Ghosh & Reddy, 2012 ▸; Reddy *et al.*, 2010 ▸; Sun & Hou, 2008 ▸; Feng & Grant, 2006 ▸; Reddy *et al.*, 2005 ▸; Panda *et al.*, 2015 ▸).

### 3.2. BF_2_dbm(OMe)_2_   

**Figure 3:**
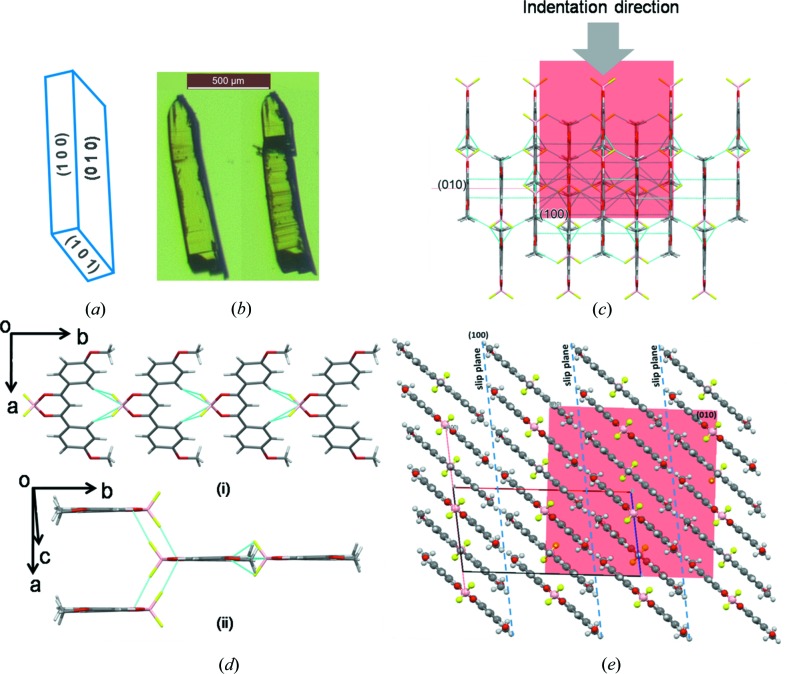
Crystal packing in BF_2_dbm(OMe)_2_. (*a*) Face indices. (*b*) Crystal before (left) and after (right) mechanical shearing deformation. (*c*) Showing molecular arrangement with respect to the indentation direction (grey arrow). (*d*), (i) one-dimensional tape formed by C—H⋯(BF_2_O_2_). (ii) Partial representation of a two-dimensional sheet (molecules viewed from side). (*e*) Representation of the slip planes in crystal packing and the orientation of the indentation direction with respect to slip planes; the (010) plane (on which indentation has been done) is shown in red.

BF_2_dbm(OMe)_2_ is known to crystallize in the centrosymmetric monoclinic space group *C*_2_/*c*, with half a molecule in the asymmetric unit (Fig. S1(ii), for clarity the full molecule is shown), which is redetermined here (Fig. 3 ▸) (Yoshii *et al.*, 2013 ▸). The bifurcated C—H⋯F (2.67 Å, 157.56°) and C—H⋯B (3.019 Å, 162.24°) intermolecular interactions between the phenyl and BF_2_O_2_ groups form linear tapes, which are further linked by additional C—H⋯F interactions (2.57 Å, 118.26°) to form two-dimensional sheets. In the crystal packing slip planes are formed by —OCH_3_ functional groups. In this case the crystals undergo plastic shearing deformation (Ghosh & Reddy, 2012 ▸; Reddy *et al.*, 2010 ▸; Krishna *et al.*, 2015 ▸; Bag *et al.*, 2012 ▸) and do not show plastic bending on any of the faces.

### 3.3. BF_2_dbmOMe   

**Figure 4:**
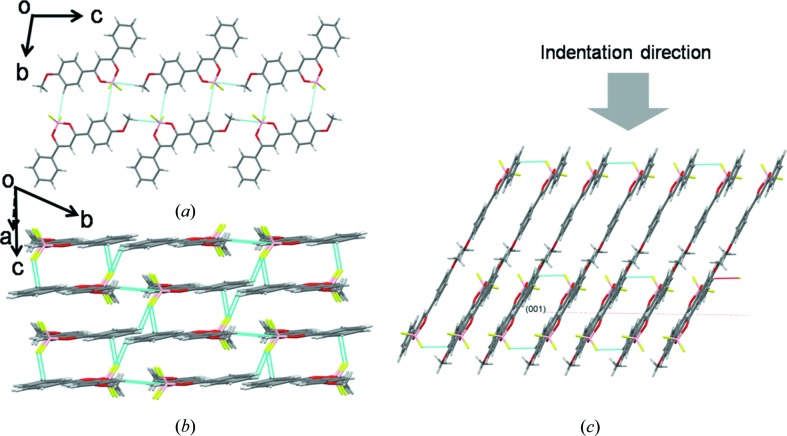
Crystal packing in BF_2_dbmOMe. (*a*) One-dimensional tape formed by C—H⋯(BF_2_O_2_). (*b*) Side view of the molecules to show three-dimensional interlocking *via* multiple hydrogen bonds. (*c*) Showing the indentation direction (grey arrow) with respect to crystal packing.

BF_2_dbmOMe crystallizes in triclinic 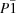 with one molecule in the asymmetric unit (Fig. S1(iii)). Unlike the other two compounds, the molecule here does not possess mirror symmetry, because the hydrophobic —OCH_3_ functional group is substituted only on one phenyl ring and not on both sides. Notably, the lone —OCH_3_ functional group fails to form the slip planes in this structure. Instead, the H atom of the —OCH_3_ group forms a C—H⋯B (3.174 Å, 159.78°) interaction (shorter than the sum of van der Waals radii, 3.28 Å) with the BF_2_O_2_ group (Alemany *et al.*, 2014 ▸). Molecules are further linked by multiple C—H⋯F interactions leading to the three-dimensional interlocking of the structure (Fig. 4 ▸ *b*). As a result the crystals show brittle mechanical behaviour (Reddy, Krishner *et al.*, 2006 ▸; Reddy, Padmanabhan *et al.*, 2006 ▸; Ghosh & Reddy, 2012 ▸; Reddy *et al.*, 2010 ▸; Sun & Hou, 2008 ▸; Feng & Grant, 2006 ▸).

## 4. Mechanical properties of molecular crystals   

The qualitative mechanical deformation experiments performed on the crystals of three compounds revealed that their mechanical behaviour is distinct from each other. Even though the three compounds contain a common backbone, *i.e.* BF_2_dbm, the different substituent hydrophobic functional groups lead to unique crystal packing arrangements in them. The molecular crystals of BF_2_dbm(^*t*^Bu)_2_ deformed plastically when bent on the (001) crystal face and the crystal packing is consistent with the established bending model, *i.e.* the existence of anisotropy in the crystal packing in such a way that the strong and weak interactions are arranged in nearly perpendicular directions (Reddy *et al.*, 2005 ▸; Reddy, Padmanabhan *et al.*, 2006 ▸). Furthermore, the molecules are connected *via* multiple weak C—H⋯F intermolecular interactions in two directions, but in the third direction the adjacent sheets pack *via* only the ^*t*^Bu functional groups (Fig. 2 ▸ *c*). Thus, it is not surprising that the crystals plastically bend upon the application of a mechanical stress. In the case of BF_2_dbm(OMe)_2,_ the molecular crystals undergo inhomogeneous shearing deformation, as shown in Fig. 1 ▸. Typically, the observation of such a deformation mode indicates that some specific crystallographic planes offer very low resistance to shearing upon the application of stress, whereas the other planes exhibit considerably more resistance. Therefore, plastic deformation is restricted only to those ‘easy sliding’ molecular planes. This is consistent with the crystal packing features wherein the molecules are packed into flat two-dimensional layers with the support of C—H⋯F interactions. The —OCH_3_ functional groups close pack to form slip planes. Here the two-dimensional layers, comprising molecules between the slip planes (see Fig. 3 ▸ *e*), are thick and hence are not ideal to slide one over the other to result in the easy shearing deformation. However, on application of a mechanical stress the layers slide to some extent and create striations on the crystal due to inhomogeneous shearing (Fig. 1 ▸). Further application of the mechanical stress leads to fracture of the crystal. As there are slip planes in the structure we also tried to bend them, but did not show any plastic bending nature. This is typically the case when the shear stress is comparable to or exceeds the fracture stress (Ghosh *et al.*, 2013 ▸). In the molecular crystals of BF_2_dbmOMe, three-dimensional interlocked packing *via* the C—H⋯(BF_2_O_2_) and C—H⋯F intermolecular interactions do not allow for plastic deformation and hence fail in a brittle manner.

**Figure 5:**
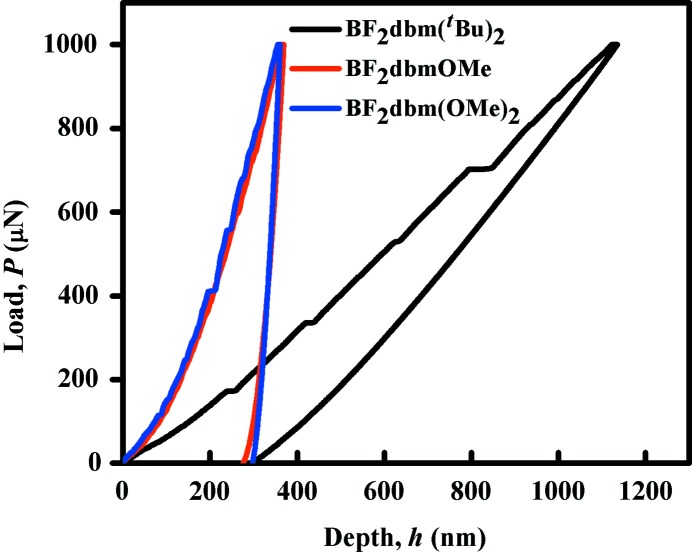
Representative *P*–*h* curves obtained from the molecular crystals of BF_2_dbm(^*t*^Bu)_2_ (black line), BF_2_dbm(OMe)_2_ (blue line) and BF_2_dbmOMe (red line).

**Figure 6:**
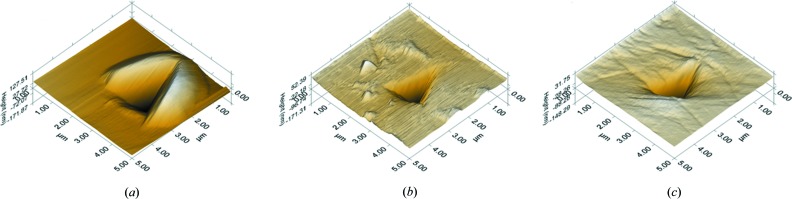
The AFM images of the residual indent impressions of (*a*) BF_2_dbm(^*t*^Bu)_2_, (*b*) BF_2_dbm(OMe)_2_ and (*c*) BF_2_dbmOMe. A considerable pile-up of material around the indent in (*a*) indicates the soft nature of the crystal. No evidence of cracking was observed in any of the indents.

*Quantification of mechanical properties by nanoindentation (NI) experiments*: Recent work has successfully demonstrated that the NI technique can be utilized to quantify the mechanical properties of molecular crystals, and in turn not only establish the structure–property correlations, but also use such knowledge for designing organic solids with specific targeted properties. The major faces of the crystals of BF_2_dbm(^*t*^Bu)_2_, BF_2_dbm(OMe)_2_ and BF_2_dbmOMe were all indented in load-control mode with a Berkovich tip, and (un)loading rates of 0.2 mN s^−1^, peak load, *P*_max_, of 1 mN, and *P*_max_ hold time of 2 s. Representative load, *P*, *versus* depth, *h*, curves are displayed in Fig. 5 ▸, which reveal the following significant differences in the mechanical responses of the three compounds examined.

(*a*) The loading part of the *P*–*h* curve obtained on BF_2_dbmOMe is smooth, whereas the corresponding ones obtained on BF_2_dbm(OMe)_2_ and BF_2_dbm(^*t*^Bu)_2_ are serrated with several discrete displacement bursts (or ‘pop-ins’). Prior work on nanoindentation of molecular crystals has shown that such displacement jumps, *h*_pop-in_, associated with the pop-ins are integer multiples of the relevant inter-planar spacing of the crystal, typically the slip plane. In the case of BF_2_dbm(^*t*^Bu)_2_, the observed *h*_pop-ins_ are multiple integers of ∼ 5 nm, which correspond to ∼ 10 times the *d*-spacing (0.0502 nm) of the slip planes. In the case of BF_2_dbm(OMe)_2_, the corresponding *h*_pop-in_ is ∼ 5.4 nm, which again is an integer multiple of 0.3541 nm (about 15 times).

(*b*) The *P*–*h* responses obtained on BF_2_dbm(OMe)_2_ and BF_2_dbmOMe are nearly identical (in contrast to the distinct shearing and brittle behaviour in qualitative tests, respectively), except for the serrations in the former’s loading curve. Indeed, the elastic modulus, *E*, and hardness, *H*, values extracted from these *P*–*h* curves using the standard Oliver–Pharr (O–P) method are also similar (see Table 1 ▸). Further, the indentation impressions are also similar (see Fig. 6 ▸), with no significant pile-up around the indents. The response obtained from BF_2_dbm(^*t*^Bu)_2_, in contrast, is significantly different. First, for the same *P*_max_, the maximum depth of penetration, *h*_max_, is significantly larger. Yet, the residual depth upon complete unloading is nearly the same in all the three crystals. This suggests that BF_2_dbm(^*t*^Bu)_2_ is significantly softer and compliant (as indicated by the high rate of elastic recovery during unloading), which is confirmed by the relatively smaller *E* and *H* values extracted from the *P*–*h* curves using the O–P method. Here it is important to note that significant pile-up around the indent (see Fig. 6 ▸ *a*) is noted, which indicates the ease of plastic flow. However, the pile-up affects the accuracy of estimated *E* and *H* values, as the effective contact area altered significantly. In view of this, we will not utilize these quantitative metrics in further discussion, except to note that BF_2_dbm(^*t*^Bu)_2_ is soft and compliant compared with the other two compounds examined in this work.

Understanding the above observations, made on the NI results, requires the consideration of crystal packing with respect to the indentation direction. The qualitative tests indicate that BF_2_dbm(^*t*^Bu)_2_, BF_2_dbm(OMe)_2_ and BF_2_dbmOMe crystals are bending, shearing and brittle types, respectively. The NI experiments indeed confirm that BF_2_dbm(^*t*^Bu)_2_ is much softer (plastic) vis-à-vis the other two. As mentioned earlier, the presence of slip planes orthogonal to relatively stronger interactions in the crystal packing promotes plasticity (Reddy *et al.*, 2005 ▸; Reddy, Padmanabhan *et al.*, 2006 ▸; Panda *et al.*, 2015 ▸). In BF_2_dbm(^*t*^Bu)_2_ crystals, (100) formed by the hydrophobic ^*t*^Bu groups (Fig. 2 ▸ *c*) is the slip plane. This allows for easy plastic deformation when the crystals are stressed through bending upon the (001) face that is orthogonal to the slip plane. The NI experiments, which were also performed on the (001) plane, *i.e.* the major face (Fig. S4*a*). This means that the indentation direction [001] is parallel to the slip planes (*bc*-plane). Moreover, the indentation direction is oblique to the direction of the π-stacked molecules (Fig. 2 ▸ *c*). These geometrical factors favour easier shearing of the molecular layers during deformation. Hence, the indenter penetrates easily into the crystal, giving rise to low *H*.

In the case of BF_2_dbm(OMe)_2_, while the qualitative experiments reveal its suceptibility to localized plastic shearing, the NI experiments yield high *E* and *H* values, implying high resistance to elastic and plastic deformations. These seemingly contradictory results can be rationalized as follows. We could perform the NI experiments only on the major face (010) of single crystals of BF_2_dbm(OMe)_2_ (Fig. S4*b*). In this crystal structure, the (100) planes are the slip planes, and also parallel to the indentation direction of [010]. However, here along this direction molecules are linked by multiple and strong C—H⋯F interactions compared with the weak π-stacking interactions in BF_2_dbm(^*t*^Bu)_2_ (see the orientation of molecules in Figs. 2 ▸ *c* and 3 ▸ *e*). The superior restorative nature of the C—H⋯F interactions produces higher *E* and *H* values in this case than for the other two samples. In this context it is worth noting that the crystal deformation is highly influenced by molecular arrangement and directional and/or non-directional interactions in the particular indentation direction (Varughese *et al.*, 2013 ▸; Kiran *et al.*, 2010 ▸; Varughese *et al.*, 2012 ▸; Kiran, Varughese *et al.*, 2013 ▸). Desiraju and coworkers state that ‘*the short-range nondirectional interactions (such as van der Waals interactions) are likely to influence the plastic response as they will have a large bearing on how bonds break, whereas directional interactions (such as hydrogen bonds), being effective at long separations, influence elasticity because of their restorative character*’ (Varughese *et al.*, 2013 ▸). Further, we recognize in this context that it would have been better if we had indented the crystal on other facets as well, so as to obtain a good idea on the anisotropy in plastic properties. Unfortunately, the small size of the crystals precluded us from pursuing this matter to its logical end.

The *E* and *H* values of the third compound, BF_2_dbmOMe, were found to be slightly lower than the respective values for BF_2_dbm(OMe)_2_. In this case, indentation experiments were performed on the major face (001) of the crystals. Notably, the orientation of molecules along the indentation direction is similar to that of BF_2_dbm(OMe)_2_. Indeed, the C—H⋯F interactions are slightly oblique to the indentation direction, which is probably the reason for the slightly lower *E* and *H* than that of BF_2_dbm(OMe)_2_. The —OCH_3_ functional groups in this structure do not form the slip planes, which would otherwise facilitate plastic flow. This makes stress relaxation in this crystal difficult and hence the crystals exhibit brittle behaviour, which is consistent with its three-dimensional interlocked structure with more contributions from directional interactions (Fig. S5) and the absence of slip planes in it.

## 5. Mechanochromic luminescence experiments   

Experiments to examine the ML characteristics of the three compounds were performed by taking a few single crystals of the respective compounds (separately) in a mortar and gently crushing with a pestle to form a thin layer of powder particles. Firm mechanical stress was applied on the resulting thin powder layers and the prominence of mechanochromic luminescence was examined under UV light (365 nm). The bending type BF_2_dbm(^*t*^Bu)_2_ crystals exhibited prominent colour changes from cyan (450 nm) to yellow (548 nm), while a reasonably good change in emission colour from green to yellow (only broadening of the peak) was observed for the shearing type BF_2_dbm(OMe)_2_ even at room temperature (Figs. 1 ▸ and S3). Moreover, both the materials healed back to their original colour, slow at room temperature but more quickly upon heating with a hot air gun. However, the third compound, BF_2_dbmOMe, did not exhibit any perceptible colour change at room temperature as well as at −98°C, which was obtained by immersing the mortar into frozen methanol using liquid N_2_ solution. This is in good agreement with the observed qualitative mechanical properties. The results support our earlier hypothesis that the plastic deformation behaviour effects the ML behaviour of solid-state fluorophores. The quantitative NI data does not allow ranking of plasticity in the three solids, which is mainly because of the absence of the data from the other observed faces.

The reversibility of ML behaviour depends on the plastic/elastic nature of the material. Longer recovery times are required for a material with high plastic behaviour. These observations are further supported by the elastic recovery rates of the *P*–*h* curves of the three compounds obtained from the NI experiments. BF_2_dbm(OMe)_2_ and BF_2_dbmOMe molecular crystals gave a higher elastic recovery rate than for BF_2_dbm(^*t*^Bu)_2_ crystals. In addition to that, the perturbed yellow states on recovery emit the same colour as their parent forms, which suggests that the perturbed states must remain close enough to their original solid-state structure, but with defects that allow some changes in the molecular environment so that they return to the same form with time or on heating (Krishna *et al.*, 2013 ▸). The powder X-ray diffraction analysis also suggests that the recovery of the samples subjected to mechanical grinding is much slower in the case of BF_2_dbm(^*t*^Bu)_2_ and BF_2_dbm(OMe)_2_ compared with the BF_2_dbmOMe samples. As expected, the recovery of the brittle BF_2_dbmOMe samples is so fast that it does not show any significant decrease in the intensities of the peaks upon ball milling for 30 min due to partial amorphization of the material, while the peaks in both bending type BF_2_dbm(^*t*^Bu)_2_ and shearing type BF_2_dbm(OMe)_2_ show a significant decrease, confirming their superior plasticity than that of the former. On the other hand, the solid-state emission spectra of BF_2_dbm(^*t*^Bu)_2_ showed a significant red shift (from 450 to 548 nm) as well as broadening of the emission band upon mechanical grinding (FWHM from 41 to 115 nm), while BF_2_dbm(OMe)_2_ showed only a slight broadening of the emission band (FWHM from 56 to 77 nm), but no shift was observed in the maxima (Fig. S3). No change was observed in the case of BF_2_dbmOMe. These results are also in good agreement with the recovery dynamics of the three samples, expected from their plasticity order.

## 6. Conclusions   

We have examined the mechanical properties of three compounds, namely BF_2_dbm(^*t*^Bu)_2,_ BF_2_dbm(OMe)_2_ and BF_2_dbmOMe crystals, using qualitative mechanical deformation tests and quantitative analysis using nanoindentation experiments. The preliminary qualitative tests revealed that BF_2_dbm(^*t*^Bu)_2_, BF_2_dbm(OMe)_2_ and BF_2_dbmOMe crystals undergo bending, shearing and brittle deformation, respectively. Further, NI experiments confirmed that the BF_2_dbm(^*t*^Bu)_2_ crystals are much softer with the lowest elastic modulus (0.369 ± 0.008 GPa) and hardness (92.45 ± 4.04 MPa) values compared with both shearing (BF_2_dbm(OMe)_2_) and brittle (BF_2_dbmOMe) type crystals. The observed mechanical properties in all the cases were consistent with their underlying crystal packing. In addition to that, BF_2_dbm(^*t*^Bu)_2_ and BF_2_dbm(OMe)_2_ molecular crystals showed a prominent ML property from cyan to yellow and green to yellow, respectively, due to their higher plasticity, whereas BF_2_dbmOMe molecular crystals did not. After analyzing the results of these three examples, we conclude that the ML behaviour positively correlates with the plasticity. Hence introducing the slip planes into the crystal packing by keeping hydrophobic functional groups such as ^*t*^Bu and —OCH_3_ as substituents can help the design of efficient ML materials.

## 7. Experimental   

BF_2_dbm(^*t*^Bu)_2_, BF_2_dbm(OMe)_2_ and BF_2_dbmOMe compounds were synthesized according to previously reported procedures (Karasev & Korotkich, 1986 ▸; Yoshii *et al.*, 2013 ▸; Sun *et al.*, 2012 ▸; Zawadiak & Mrzyczek, 2012 ▸) and all the reagents and solvents were received from Aldrich chemicals which were used as such without any further purification. All the compounds were crystallized from commercially available solvents by the slow evaporation method at ambient conditions. BF_2_dbm(^*t*^Bu)_2_ single crystals obtained from hexane:ethyl acetate (1:1 ratio), BF_2_dbm(OMe)_2_ and BF_2_dbmOMe were obtained from acetone solvent. After 2–3 days the crystals were suitable for single-crystal X-ray diffraction (SCXRD) experiments as well as for studying mechanical properties by: (i) qualitative experiments using metal forceps and a needle under the microscope and (ii) quantitative experiments using the NI technique. Initially, good quality crystals were selected under the microscope and face indexing experiments were carried out to identify the faces of all the three crystals and then used for NI experiments.

For indentation experiments, single crystals were firmly mounted on a stud using cyanoacrylate glue. Experiments were performed on each major facet of the three compounds using a nanoindenter (Triboindenter of Hysitron, Minneapolis, USA) with an *in situ* imaging capability. The machine continuously monitors and records the load, *P*, and displacement, *h*, of the indenter with force and displacement resolutions of 1 nN and 0.2 nm, respectively. A three-sided pyramidal Berkovich diamond indenter (tip radius ∼ 100 nm) was used to indent the crystals. Loading and unloading rates of 0.2 mN s^−1^ and a hold time of 2 s at peak load were employed. In order to identify flat regions for the experiment, the crystal surfaces were imaged prior to indentation using the same indenter tip. A minimum of 10 indentes were performed on each crystallographic face to ensure reproducibility. The indentation impressions were captured immediately after unloading so as to avoid any time-dependent elastic recovery of the residual impression. The *P*–*h* curves obtained were analyzed using the standard Oliver–Pharr method (Oliver & Pharr, 1992 ▸) to extract the elastic modulus, *E*, of the crystal in that orientation and the detailed methodology is given elsewhere (Bolshakov *et al.*, 1996 ▸). However, this method was not used where the pile-up is found around the indenter.

### 7.1. Crystallography   

Crystals of all three compounds were individually mounted on a glass pip. Intensity data were collected on a Bruker KAPPA APEX II CCD Duo system with graphite-monochromatic Mo *K*α radiation (λ = 0.71073 Å). The data were collected at 100 K and the data reduction was performed using Bruker *SAINT* software (Bruker, 2003 ▸). Crystal structures were solved by direct methods using *SHELXL*97 and refined by full-matrix least-squares on *F* ^2^ with anisotropic displacement parameters for non-H atoms using *SHELXL*97 (Bruker, 2000 ▸). H atoms associated with C atoms were fixed in geometrically constrained positions. H atoms associated with O and N atoms were included in the located positions. Structure graphics shown in the figures were created using the *X-Seed* software package Version 2.0.10.

### 7.2. Differential scanning calorimetry (DSC)   

DSC was conducted on a Mettler–Toledo DSI1 STAR^e^ instrument. Accurately weighed samples (3–4 mg) were placed in hermetically sealed aluminium crucibles (40 µL) and scanned from 30 to 300°C at a heating rate of 5°C min^−1^ under a dry nitrogen atmosphere (flow rate 80 ml min^−1^). The data were managed by *STAR* ^e^ software (Fig. S2*d*).

### 7.3. Powder X-ray diffraction (PXRD)   

The PXRD patterns were collected on a Rigaku SmartLab with a Cu *K*α radiation (1.540 Å). The tube voltage and amperage were set at 20 kV and 35 mA, respectively. Each sample was scanned between 5 and 50° 2θ with a step size of 0.02° (Fig. S2). The instrument was previously calibrated using a silicon standard.

### 7.4. Solid state UV–vis absorption and emission spectra   

Spectra were collected using a JASCO V-670 spectrophotometer and a Horiba Jobin Yvon Fluorolog CP machine (USA), iHR 320 model spectrometer equipped with 450 W Xe lamp, respectively. Initially both absorption and emission spectra were recorded for a smoothly smeared powder sample (unground), after that the sample was gently ground for 5 min with a mortar and pestle, and both the spectra were immediately recorded (the same procedure was repeated for all three compounds; Fig. S3).

## Supplementary Material

Crystal structure: contains datablock(s) global, bf2dbmome2_a, bf2dbm_ome, bf2dbmtbu2_a. DOI: 10.1107/S2052252515015134/ed5006sup1.cif

Structure factors: contains datablock(s) bf2dbmome2_a. DOI: 10.1107/S2052252515015134/ed5006bf2dbmome2_asup2.hkl

Structure factors: contains datablock(s) bf2dbm_ome. DOI: 10.1107/S2052252515015134/ed5006bf2dbm_omesup3.hkl

Structure factors: contains datablock(s) bf2dbmtbu2_a. DOI: 10.1107/S2052252515015134/ed5006bf2dbmtbu2_asup4.hkl

Supporting tables and figures. DOI: 10.1107/S2052252515015134/ed5006sup5.pdf

CCDC references: 1418732, 1057686, 1057687

## Figures and Tables

**Table 1:** Hardness (*H*) and elastic modulus (*E*) values obtained from the major faces of the crystals of BF_2_dbm(^*t*^Bu)_2_, BF_2_dbm(OMe)_2_ and BF_2_dbmOMe

Sample	Crystal face	Slip plane	Elastic modulus, *E* (GPa)	Hardness, *H* (MPa)	Crystal density (gcm^3^)	m.p. (C)
BF_2_dbm(^*t*^Bu)_2_	(001)	(100)	0.3690.008	92.454.04	1.545	257.7
BF_2_dbm(OMe)_2_	(010)	(100)	10.8640.249	264.9310.98	1.603	239.0
BF_2_dbmOMe	(001)	No specific plane	8.6200.176	255.798.48	1.482	221.3
