# Supplementary material for: Structure–mechanical property correlations in mechanochromic luminescent crystals of boron difluoride dibenzoylmethane derivatives
Source: IUCrJ. 2015 Sep 22;2(Pt 6):611–9. doi: 10.1107/S2052252515015134 (PMC4645105; doi:10.1107/S2052252515015134)
Supplement: Supplementary file 5 [file m-02-00611-sup5.pdf]

**IUCrJ**

**Volume 2 (2015)**

**Supporting information for article:**

# Structure–mechanical property correlations in mechanochromic luminescent crystals of boron difluoride dibenzoylmethane derivatives

**Gamidi Rama Krishna, Ramesh Devarapalli, Rajesh Prusty, Tiandong Liu, Cassandra L. Fraser, Upadrasta Ramamurty and Malla Reddy Chilla**

**Table S1** Crystallographic data and structure refinement parameters

| | **$BF_2$dbm(tBu)$_2$** | **$BF_2$dbm(OMe)$_2$*** | **$BF_2$dbmOMe** |
|---|---|---|---|
| Formula | $C_{23}H_{27}BF_2O_2$ | $C_{17}H_{15}BF_2O_4$ | $C_{16}H_{13}BF_2O_3$ |
| Crystal System | monoclinic | monoclinic | Triclinic |
| Space group | *C2/c* | *C2/c* | *P-1* |
| *a* [ Å ] | 28.575(4) | 21.1854(15) | 8.0234(7) |
| *b* [ Å ] | 7.0402(9) | 7.0826(5) | 9.0901(8) |
| *c* [ Å ] | 10.3208(13) | 10.0747(7) | 10.7916(8) |
| $\alpha$ [°] | 90 | 90 | 75.514(7) |
| $\beta$ [°] | 102.920(3) | 98.208(2) | 80.766(7) |
| $\gamma$ [°] | 90 | 90 | 69.797(8) |
| $V$ [Å $^3$] | 2023.7(5) | 1496.20(18) | 712.79(11) |
| $Z$ | 4 | 4 | 2 |
| $\lambda$ [ Å ] | 0.71073 | 0.71073 | 0.71073 |
| $\rho_{calcd}$ [gcm$^{-3}$] | 1.2611 | 1.474 | 1.407 |
| $F$[000] | 816.5 | 688.0 | 312.0 |
| $\mu$ [mm$^{-1}$] | 0.090 | 0.119 | 0.112 |
| $2\theta$ [°] | 2.92 to 55.98 | 3.88 to 56 | 3.92 to 56.24 |
| index ranges | $-41 \le h \le 41$<br>$-10 \le k \le 10$<br>$-7 \le l \le 15$ | $-27 \le h \le 27$<br>$-9 \le k \le 9$<br>$-13 \le l \le 11$ | $-10 \le h \le 10$<br>$-9 \le k \le 11$<br>$-14 \le l \le 12$ |
| $T$ [K] | 100 | 100 | 100 |
| $R$1 | 0.0348 | 0.0327 | 0.0566 |
| $wR$2 | 0.0946 | 0.0924 | 0.1235 |
| $R_{merge}$ | 0.0413 | 0.0359 | 0.0779 |
| Parameters | 131 | 112 | 200 |
| GOF | 1.055 | 1.081 | 1.040 |
| reflns total | 21860 | 13225 | 4141 |
| unique reflns | 2442 | 1811 | 2991 |
| obsd reflns | 2124 | 1657 | 2092 |
| ccdc/Refcode | 1057664 | 905565/SANKUO02 | 1057663 |

* This is from our redetermined data. The REFCODE for the reported structure is SANKUO02 (Yoshii *et al*., 2013. in the main text).

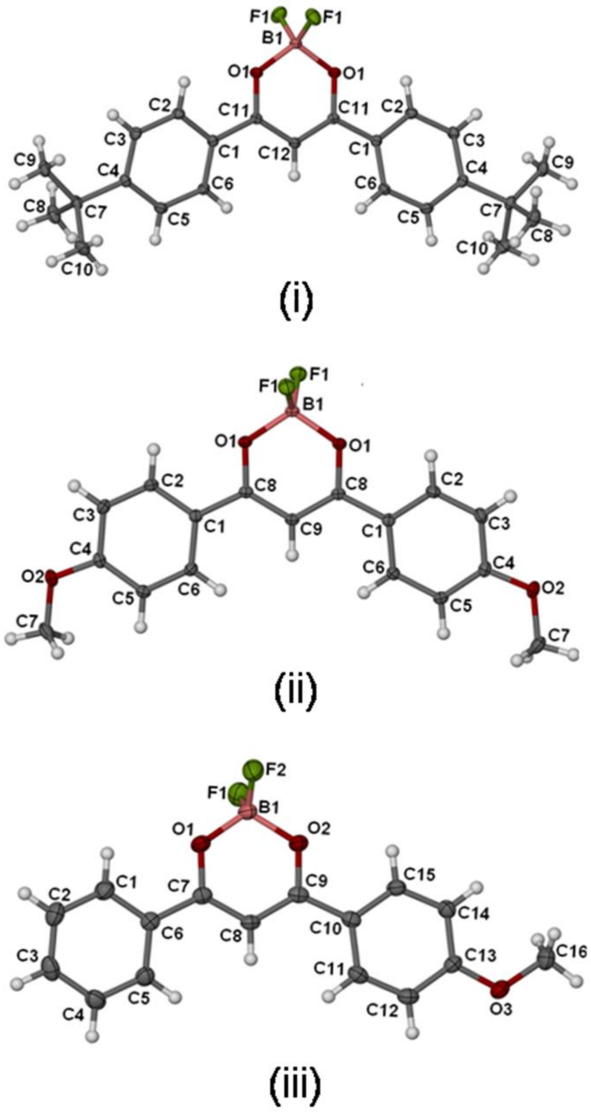


**Figure S1** ORTEP representation of (i) $BF_2$dbm(*t*Bu)$_2$, (ii) $BF_2$dbm(OMe)$_2$, and (iii) $BF_2$dbmOMe respectively. Displacement ellipsoids are drawn at the 50% probability level.

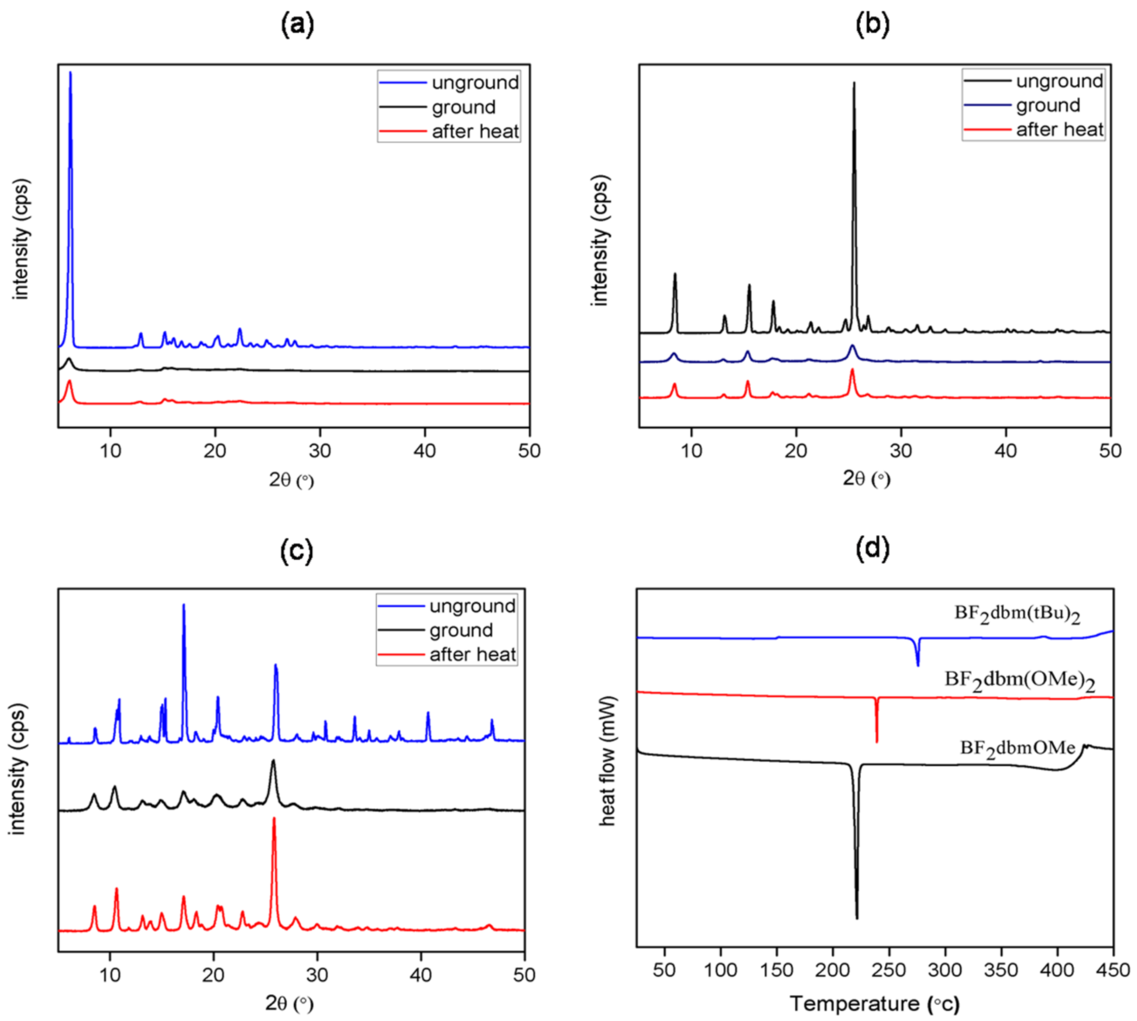


**Figure S2** (a), (b) and (c) shows the comparison of powder x-ray diffraction patterns of $BF_2$dbm(*t*Bu)$_2$, $BF_2$dbm(OMe)$_2$ and $BF_2$dbmOMe samples in various states respectively (before grinding (blue line), after 30min ball mill grinding (black line) and after heating the ground sample (red line)). (d) DSC curves of respective compounds.

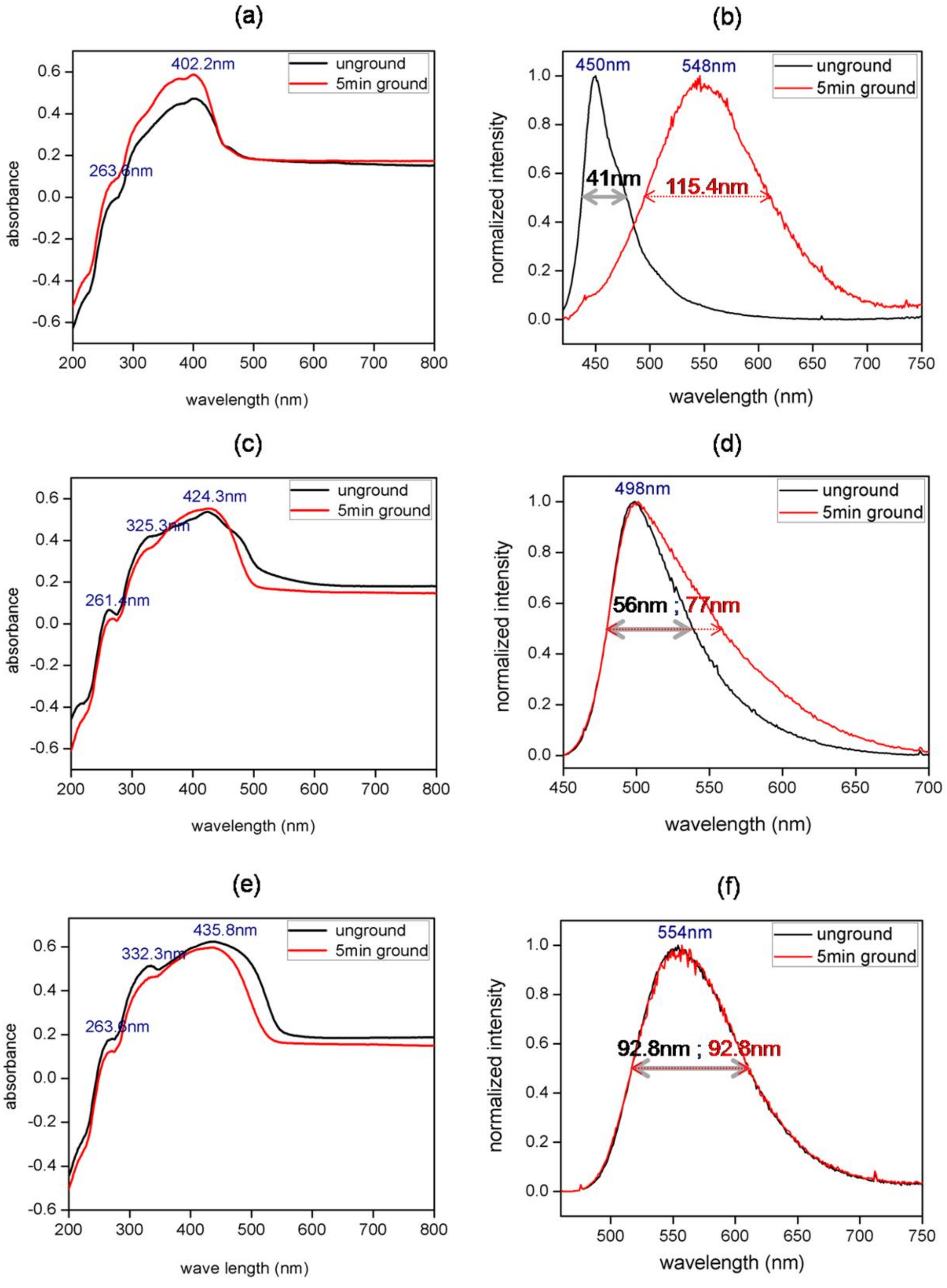


**Figure S3** Solid state absorbance and emission spectra: (a), (b) corresponds to $BF_2dbm(tBu)_2$; (c), (d) corresponds to $BF_2dbm(OMe)_2$; and (e), (f) corresponds to $BF_2dbmOMe$ compounds respectively. Note: in all the graphs black color line represents unground sample and red color line represents 5min ground sample (using a mortar and pestle). All the emission spectra were recorded at the excitation of corresponding highest absorption maxima.

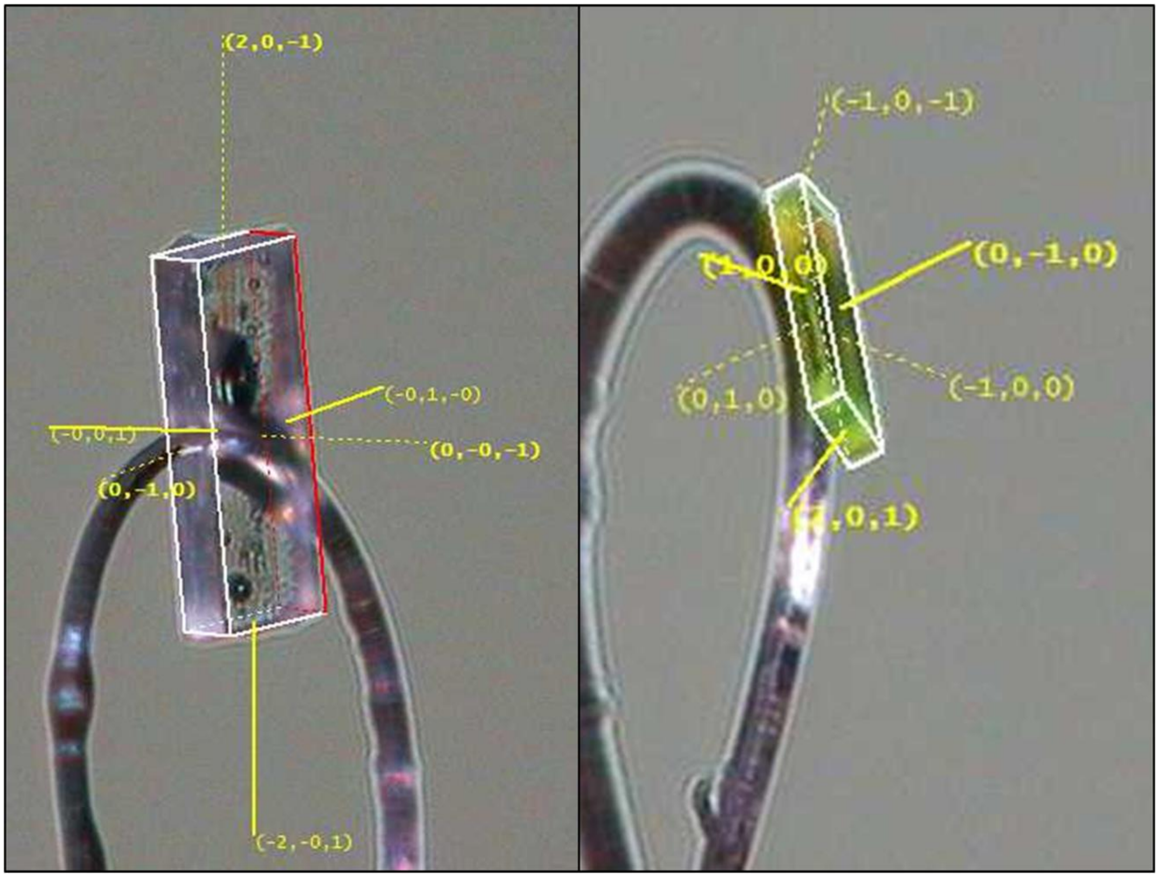


**Figure S4** Face index images of $BF_2$dbm(*t*Bu)$_2$, $BF_2$dbm(OMe)$_2$.

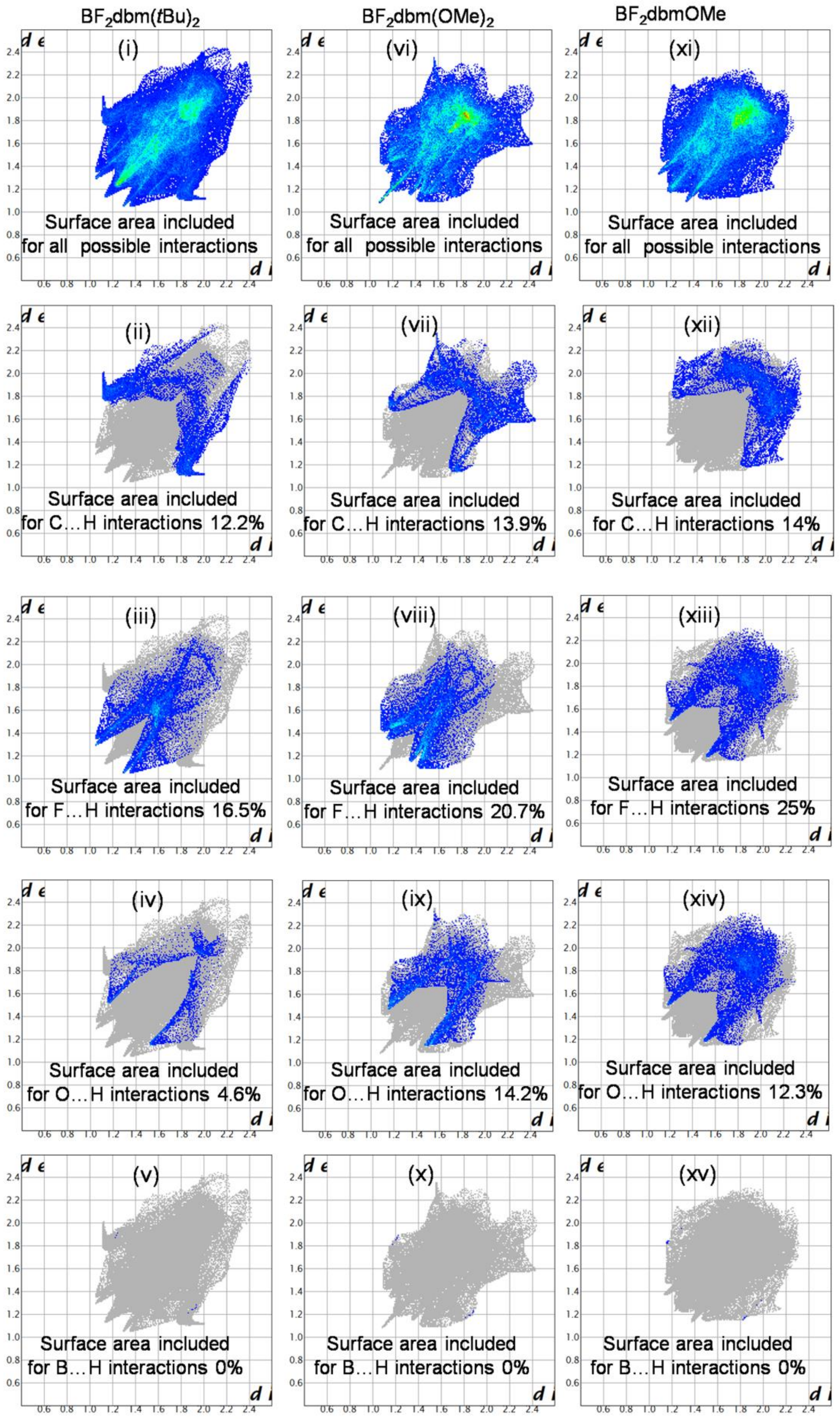


**Figure S5** Hirshfeld fingerprint plots for various interactions of all three compounds.
